# Comparing Traditional and Digitized Cognitive Tests Used in Standard Clinical Evaluation – A Study of the Digital Application Minnemera

**DOI:** 10.3389/fpsyg.2019.02327

**Published:** 2019-10-18

**Authors:** Stina Björngrim, Wobbie van den Hurk, Moises Betancort, Alejandra Machado, Maria Lindau

**Affiliations:** ^1^Department of Psychology, University of Stockholm, Stockholm, Sweden; ^2^Mindmore AB, Stockholm, Sweden; ^3^Department of Clinical Psychology, Psychobiology and Methodology, Faculty of Psychology, University of La Laguna, Tenerife, Spain; ^4^Division of Clinical Geriatrics, Center for Alzheimer Research, Department of Neurobiology, Care Sciences and Society, Karolinska Institutet, Stockholm, Sweden

**Keywords:** neuropsychology, cognition, screening battery, digitized assessment, validity, equivalence test

## Abstract

The purpose of this study was to compare a new digitized cognitive test battery, Minnemera, with its correspondent traditional paper-based cognitive tests. Eighty-one healthy adults between the ages of 21 and 85 participated in the study. Participants performed the two different test versions (traditional paper-based and digitized) with an interval of four weeks between the tests. Test presentation (the order of the test versions presented) was counterbalanced in order to control for any possible test learning effects. The digitized tests were constructed so that there were only minor differences when compared to the traditional paper-based tests. Test results from the paper-based and digitized versions of the cognitive screening were compared within individuals by means of a correlation analysis and equivalence tests. The effects of demographic variables (age, gender and level of education) and test presentation were explored for each test measure and each test version through linear regression models. For each test measure, a significant correlation between traditional and digitized version was observed ranging between *r* = 0.34 and *r* = 0.67 with a median of *r* = 0.53 (corresponding to a large effect size). Score equivalence was observed for five out of six tests. In line with previous traditional cognitive studies, age was found to be the most prominent predictor of performance in all digitized tests, with younger participants performing better than older adults. Gender was the second strongest predictor, where women outperformed men in tests measuring verbal memory; men performed better than women in tests with a strong visual component. Finally, the educational level of the test subjects had an effect on executive functions, with a higher educational level linked to a better inhibition response and working memory span. This study suggests that the tests in the Minnemera cognitive screening battery are acceptably comparable to the traditional paper-based counterparts.

## Introduction

Effective cognitive screening is needed in healthcare to be able to ascertain whether a patient needs further cognitive investigation. Widely used short cognitive screening instruments in case of suspicion of dementia are the Mini-Mental State Examination (MMSE; Folstein et al., 1975) and Clock Drawing Test (Shulman and Feinstein, 2003). These instruments are, however, coarse measurements of cognitive functioning and do not capture subtle cognitive impairment (Hooijer et al., 1992). With the global aging population (Winblad et al., 2016) and with dementia medicines in clinical trials, there is a need for cognitive screening that is more sensitive to subtle cognitive impairment, which is cost-effective (Müller et al., 2017) and which can be made available to more patients who experience subjective cognitive decline. The use of digitized cognitive screening batteries for clinical purposes have been reported to lead to a possible increase in accessibility to earlier and more precise assessment as well as serial testing to evaluate treatment (Gualtieri and Johnson, 2006), and therefore have the potential to ascertain earlier assessment, earlier diagnosis and, eventually, better prognosis. Digitized cognitive screening has been proposed as a method to track clients’ health status and support health workers in decision making (World Health Organization [WHO], 2019).

There is a growing body of literature in the field of digitized cognitive testing (García-Casal et al., 2017; Koo and Vizer, 2019). Results of previous studies of traditional versus digitized instruments are mixed (Noyes and Garland, 2008). Some of these results pertain to studies from the 1980s and 1990s which generally favored traditional paper-based tests, and mainly highlighted the visual fatigue associated with computerized tests (for a systematic review see Dillon, 1992). High-resolution displays were found to improve performance and reduce visual fatigue, compared to low-resolution displays, however, reading on hard-copy was still found to be superior (Ziefle, 1998; Wästlund et al., 2005). Further, Wästlund et al. (2005) reported higher levels of perceived stress when using a computer-based resource compared to a paper-based resource, due to higher cognitive workload, possibly affected by the quality of the displays and by the experience of using digital tools. Studies from the 2000s and onward reported reduced differences between traditional and digitized test performance, which Noyes and Garland (2008) concluded was due to technological development that has vastly improved screen quality, and that digital tools have become more and more common in everyday life, especially amongst the younger generations (Noyes and Garland, 2008).

Several digitized versions of original paper-based cognitive tests have been previously constructed and compared. For example, a digitized version of the Rey Auditory Verbal Learning Test (RAVLT; Rey, 1964; Schmidt, 1996) was studied by Morrison et al. (2018). The RAVLT test measures learning, short-term recall and long-term recall, were found to correlate moderately to strongly between test versions (traditional paper-based or digitized tests) in healthy controls and subjects with mild cognitive impairment (Morrison et al., 2018). A similar conclusion was reached by Brunetti et al. (2014) who examined the Corsi Block-tapping task. Besides finding equivalent results for Corsi in digitized compared to paper-based test versions, Brunetti et al. (2014) emphasized the additional benefits of the digitized version such as increasing the ease of administration, automation of presentation of the task, including a measurement of reaction time and automation of scoring and reacting to correct and incorrect answers. Claessen et al. (2015) compared performance on traditional Corsi with performance on a digitized version of Corsi and found small to moderate effect sizes when conducting an ANOVA for repeated measures. Paired sample *t*-tests showed that performance was significantly higher on the forward reproduction on the traditional test, compared to the digitized test. The performance on the backward reproduction was comparable between the two versions. The authors concluded that the divergent results could be explained as a result of motor priming and interference effects. Fellows et al. (2017), examined a digitized Trail Making Test (TMT) in an application on a tablet with a capacitive touch screen. Their findings indicated that the digitized version of TMT measured the same aspects of cognitive ability as the paper-based version. The authors argued that the results also provided support that the digitized version of TMT provides additional information on cognitive processes, which are unable to be provided by the paper-based version, such as detailed information on timing, pauses, and lifting of the pen. Karimpoor et al. (2017), also demonstrated similar test scores when comparing tablet-based TMT with traditional paper and pencil TMT. In another example, digitized versions of the Stroop Word and Color Test have repeatedly shown moderate to strong correlation with the results on the traditional test and their digitized counterpart (Hepp et al., 1996; DiBonaventura et al., 2010). However, the complete equivalence between traditional paper-based tests and digitized tests has also been questioned, since the different test versions were found to involve partly different cognitive processes (Dillon, 1992; Noyes and Garland, 2008). One of the most urgent issues today when it comes to digitization of neuropsychological tests is thus whether a digitized version of a test has the same psychometric properties as the traditional test (Gualtieri and Johnson, 2006).

Performance on cognitive tests is influenced by demographic factors such as age, gender and level of education (Leibovici et al., 1996; Jorm et al., 2004; Mungas et al., 2009), as well as learning through prior exposure to a similar test (Collie et al., 2003; Duff et al., 2007; Oliveira et al., 2014). Previous studies have shown demographic differences when using the traditional cognitive tests included in the current Minnemera screening battery. The RAVLT, for example, was affected by several moderating factors. Age was the moderating factor most frequently found in studies with increasing age resulting in declining performance on RAVLT (Query and Megran, 1983; Geffen et al., 1990; Schmidt, 1996; Van der Elst et al., 2005; Strauss et al., 2006). Gender was also reported to affect test results on RAVLT in several studies, where women tended to outperform men (Geffen et al., 1990; Strauss et al., 2006). Contrarily, the impact of the educational level on the result on RAVLT showed mixed results (Mitrushina et al., 2005).

Increasing age was also negatively associated with performance in the Trail making Test (TMT, Army Individual Test battery, 1944; Spreen and Strauss, 1998; Tombaugh, 2004; Mitrushina et al., 2005; Salthouse, 2011), the Paced Auditory Serial Addition Test (PASAT, Gronwall, 1977; Mitrushina et al., 2005; Ozakbas et al., 2016), the Victoria Stroop Test (VST, Van der Elst et al., 2005; Hankee et al., 2016) and the Corsi Block-tapping task (Kessels et al., 2000). Differences in results on Corsi based on gender have been demonstrated in studies where men outperformed women (Kessels et al., 2000; Brunetti et al., 2014). Deary and Der (2005) found no differences in results on PASAT based on gender and level of education. Studies have also shown differences in performance on the Boston Naming Test based on gender, age and level of education (Van Gorp et al., 1986; Neils et al., 1995). Generally, education has been found to have a beneficial effect on cognition in higher ages. In an evaluation of the importance of social economic status (SES), Wu et al. (2016) found a connection between higher education and higher cognitive level in a large sample of Chinese people aged 50 and over. The authors hypothesized that this relationship may be due to the larger cognitive reserves acquired by those with higher education, and by their continued preferences for intellectual activities stimulating the brain.

The digitized cognitive screening Minnemera is a collection of several well-known cognitive tests which aim to measure different cognitive domains (i.e., processing speed, attention, verbal and visual memory, and executive functions; see Table 1). Minnemera aims to distinguish between normal and non-normal test results. If Minnemera’s ability in this regard proves to be good, then the instrument could serve as a guide for clinicians in the initial assessment of the patient’s cognitive state. However, in the present study we set out from the circumstance that Minnemera was designed to be as similar to the traditional paper-based tests as possible in order to obtain initial convergent validity with the paper format. Some modifications of the tests were, however, carried out to suit a digital format. For further details, please see Supplementary Appendix A.

**TABLE 1:** List of paper-based cognitive tests grouped by cognitive functions.

**Global cognition and clinical variables**
Mini-Mental State Examination (MMSE, Folstein et al., 1975) - Swedish version (MMSE-SR-2010, Palmqvist et al., 2013).
**Processing Speed and Attention**
Paced Auditory Serial Addition Test (PASAT, Gronwall, 1977; Rudick et al., 1997)
Trail Making Test, part A (TMT A, Army Individual Test battery, 1944)
**Learning and Memory**
Rey Auditory Verbal Learning Test (RAVLT, Rey, 1964; Schmidt, 1996)
**Working Memory, Executive Functions**
Trail Making Test, part B (TMT B, Army Individual Test battery, 1944)
Corsi Block-tapping task– forward and backward (Corsi, 1972; Kessels et al., 2000)
Victoria Stroop Test – Interference effect (VST, Stroop, 1935; Regard, 1991)
**Language**
Boston Naming Test (BNT, Kaplan et al., 1983) – modified version of 15 items (BNT-15, Jørgensen et al., 2017)

The overall purpose of the study was to compare Minnemera with the traditional paper-based versions of the tests upon which it is based. This was primarily done by investigating to what degree digitized screening of cognitive ability through the Minnemera digitized cognitive screening battery was consistent with a traditional screening battery with paper-based tests and a trained test leader. Additionally, the study set out to explore the influence of age, gender and level of education on each and every test measure. The tests used in Minnemera were all well-established cognitive tests with good psychometric properties; the digitized version of the tests was expected to have similar psychometric properties. The hypotheses were that the digitized version was comparable to the paper-based version, and that the demographic influence on test results, as demonstrated in previous studies, should also be found in the current study, both in the paper-based as well as in the digitized version.

## Materials and Methods

### Participants

The recruitment of participants was achieved via digital advertising (social media) and via local newspapers. Physical advertisements (posters and flyers) were also left at retirement organizations and homes, universities for seniors, non-profit organizations, churches and libraries. Initially, 140 participants showed interest and were contacted by telephone for a short interview to check whether they met the inclusion and exclusion criteria for the study. The inclusion criteria were as follows: (1) a preserved global cognitive mental state measured by means of the MMSE (Folstein et al., 1975) score of at least 24; (2) that they were not psychology or psychiatry students; (3) an adequate visual and auditory perception; and (4) an adequate knowledge of Swedish (to a native or equivalent level). Participants were excluded from the study if they met any of the following criteria: (1) an MMSE score of less than 24; (2) a psychiatric diagnosis; (3) any neurological diseases (including a neurodegenerative disease or severe acquired brain injury, such as stroke, tumor extraction, traumatic brain injury that required hospitalization, etc.); (4) a diagnosis of any systemic diseases that could compromise cognition (Diabetes Mellitus type I, thyroid diseases, kidney disease, liver disease, hematological malignancies or ongoing cancer treatment); (5) previous long-term or current substance abuse; and (6) use of medication that could potentially affect cognitive performance.

From the 111 potential candidates that showed interest in the study, fifteen were excluded for not meeting one or more of the inclusion criteria. The rest of the participants were scheduled for testing where they were subjected to an extensive interview concerning their cognitive health, and measurement of global cognitive mental state by means of MMSE test (Folstein et al., 1975). A further fifteen participants were excluded after scheduling because they withdrew from the study, either before (nine participants) or after (six participants) their first testing appointment. The final sample consisted of 81 individuals (see demographic distribution in Table 2). The sample was selected according to the most frequent demographic variables affecting cognitive tests: age, gender and education. All participants spoke fluent Swedish, however, nine participants (11.11%) had a mother tongue other than Swedish.

**TABLE 2:** Demographic characteristics of the sample.

**Age**	**Gender**	**Education**	**n (%)**	**Age M (SD)**	**Age range**	**Test presentation (A/B), n**
≤60 years	Women	≤12 years	2 (2.5)	52.5 (2.1)	51–54	0/2
		>12 years	17 (21.0)	34.7 (10.4)	24–60	12/5
	Men	≤12 years	3 (3.7)	37.0 (20.4)	21–60	1/2
		>12 years	18 (22.2)	34.4 (10.0)	24–56	11/7
>60 years	Women	≤12 years	7 (8.6)	74.4 (5.7)	67–85	5/2
		>12 years	20 (24.7)	71.1 (4.6)	62–81	6/14
	Men	≤12 years	2 (2.5)	73.0 (2.8)	71–75	1/1
		>12 years	12 (14.8)	71.2 (5.7)	64–84	7/5

Total sample			81 (100)	53.9 (20.1)	21–85	43/38

*n, subsample size; M, mean; SD, standard deviation. Test presentation A, paper-based tests first, digitized at the second testing; test presentation B vice versa.*

Prior to participation, all participants received oral and written information regarding the purpose and procedure of the study, as well as information about its confidentiality. All participants signed a written informed consent form. The study was approved by the Regional Ethics Review Board in Stockholm (issue number 2017/2530-31).

### Material

The entire sample was evaluated with a set of well-established traditional cognitive tests (Table 1) which assessed the following cognitive domains: attention, processing speed, learning and memory, executive function, and language. Test selection was based on two major criteria: (1) tests more commonly used in Sweden and the European Federation of Neurological Societies (EFNS) country members (Maruta et al., 2011), and (2) tests available in the public domain. Any test holding copyright was examined for legal conditions. To avoid copyright infringements, modifications to the tests were made accordingly.

All of the included tests have been internationally used for several decades in clinical practice, and are well-reputed. For the Paced Auditory Serial Addition Test (PASAT, Gronwall, 1977) high sensitivity: 87% and high specificity: 69% was demonstrated in separating malingering subjects from controls (Woods et al., 2018). The differential diagnostic usefulness of the Trail Making Test, parts A and B (Army Individual Test battery, 1944) has been shown by Ashendorf et al. (2008) in an American sample encompassing normal controls, mild cognitive impairment and Alzheimer’s disease. For the detailed analysis of diagnostic classification accuracy in terms of sensitivity and specificity, see Ashendorf et al. (2008). The Rey Auditory Verbal Learning Test (RAVLT, Rey, 1964; Schmidt, 1996) has a wide range of applications. Ricci et al. (2012) reported good sensitivity and specificity for an RAVLT index in the differentiation between Alzheimer’s disease, the behavioral variant of frontotemporal dementia and normal controls. Likewise, Schoenberg et al. (2006) concluded that in an American sample the RAVLT had a good discriminative power in differentiating Alzheimer’s disease from traumatic brain injury, neoplasm, stroke, and presurgical epilepsy with left versus right sided dominance in samples ranging from 16 to 88 years of age. Good, diagnostic accuracy was also reported by Guariglia (2007) for the Corsi Block-tapping task (Corsi, 1972; Kessels et al., 2000) in a Brazilian sample. The Corsi Block-tapping task was found to show significant differences between patients with Alzheimer’s disease and control persons and between patients with moderate dementia and control persons, however not between control persons and mild dementia patients. A Czechia adaptation of the Victoria Stroop Test – Interference effect (VST, Regard, 1991) has been shown to be efficient in the diagnostics of mild cognitive impairment in Parkinson’s patients (Bezdicek et al., 2015), and the Boston Naming Test (BNT, Kaplan et al., 1983) by Jørgensen et al. (2017) to permit an acceptable differentiation between mild Alzheimer’s disease and non-patients in a Danish study of persons aged 60 years and over.

All selected tests were digitized, keeping to the original format as much as possible, including the scoring procedure; see Supplementary Appendix A for further details in test adaptation and/or modifications. As a summary, digitized versions of RAVLT, Corsi, and BNT-15 remained exactly the same as the traditional versions in both procedure and scoring, other than that participants were required to state when the task was finished by pressing a button (labeled “Done”); in other words, when they considered they had freely recalled all the words from the list (RAVLT learning trials, short-term and long-term recall), finished a visuospatial sequence (Corsi), or retrieved spontaneously the name of an object visually displayed (BNT-15). A non-verbal answer was also required for the RAVLT recognition test, where participants responded by means of a “no” or “yes” button. The remainder of the tests were slightly modified to fit the tablet’s display screen in the best possible way whilst aiming to maintain the traditional version’s appearance as far as was possible. For example, the traditional TMT version is normally presented in a vertical A4 paper size and the connections between consecutive numbers (TMT A) or numbers and letters (TMT B) are performed with a pen or pencil. The digitized adaptation required turning the original A4 sheet 90 degrees to fit into the horizontal 10.1″ screen of the tablet. Thus, even though number positions were not modified, their location in the screen’s canvas was shifted compared to the original paper presentation. Moreover, the digitized version was carried out by finger rather than by use of a pen or pencil. For both versions, the total time for completion was scored as the main outcome measure.

Regarding the original version of PASAT, where the participants must respond verbally to the sum of two consecutive numbers they hear, the digitized version was designed to allow non-verbal responses. This option required participants to evaluate whether the retrieved sum was odd or even and respond accordingly by pressing either the “odd” or “even” button. This additional step was applied to avoid interference in the tablet’s speech recognition system between the simultaneous verbal output (digit sequences) and the verbal input (individual’s oral response). The outcome measure for each version was amount correct. Similar modifications were implemented for the Victoria Stroop Test (henceforth Stroop Test). In both test versions, traditional and digitized, stimuli were visually presented. However, compared to the traditional version of the Stroop Test where responses are given orally, the digitized version required a non-verbal response by means of several button options corresponding to the word or word-colour match (“green,” “red,” “yellow” or “blue”). This resulted in a slightly different outcome measure for each version. In the paper-based version, total time was scored and used as outcome measure, while for the digitized version response time for each word was scored and the average response time for correct responses was calculated as outcome measure. In any case, both tests aimed to measure the same function: the ability to inhibit a learned response.

The cognitive screening was developed as a web-application running on a full-screen Chrome browser. It was administered by using a capacitive touchscreen with features that record detailed information such as timing, pauses, and lifting from the touchscreen. Speech recognition was used in the screening of verbal memory and language. The application was not available for private use but only on tablets provided by healthcare providers, in order to ensure a controlled environment and patient safety as test results are considered classified patient data. For the same reason, the test results have not been deposited in an open repository. A redacted version of the data, stripped of personal information, is available from the corresponding author on request.

### Procedure

A repeated measure design was used where the participants underwent two testing presentations: a paper-based and a digitized version of the same tests. Participants were counterbalanced for test presentation: (A) paper-based at first visit and digitized testing at second visit or (B) digitized version at first visit and paper-based testing on second visit, in order to control any possible learning effect (Scharfen et al., 2018). These test versions were presented with an average interval of four weeks (M = 4.15, SD = 0.99).

The paper-based testing was conducted by experienced psychologists or research staff trained for this purpose (LG, SB, and WH). The digitized version was self-administered in a touch screen tablet (10.1″ Windows). Both testing versions were carried out in the same location, with a duration of approximately 45 min each in a quiet environment adapted for this purpose.

### Statistical Analyses

The statistical analyses were performed in R version 3.5.0 (R Core Team, 2018). Descriptive analyses were carried out for the sample characterization. Test measures of the paper-based versus digitized versions were first compared using Pearson correlation coefficients (*r*) or Spearman rank correlation coefficients (*r*_*s*_), the latter when the requirements of normal distribution were not fulfilled. The correlation coefficients (*r* or *r*_*s*_) are interpreted following Cohen (1988) with 0.1, 0.3, 0.5 as cut-off scores (Bosco et al., 2015) for small, moderate and large effect sizes respectively. Secondly, the mean scores on the paper-based and digitized test measures were directly compared. For each test measure a *t*-test was performed to assess if there were any statistical differences between the scores, i.e., an evaluation of no difference/difference (H0: difference is zero; H1: difference is not zero) and an equivalence test was performed for each test measure to assess statistical inequivalence/equivalence between the scores (H0: difference lies beyond equivalence bounds; H1: difference lies between equivalence bounds). The lower and upper equivalence bounds were set to −1SD and +1SD, in which the SD was calculated from the paper-based result of the test measure. The equivalence test was performed following Lakens et al. (2018).

Exploratory multiple linear regression models were used to evaluate, compare, and contrast the prediction of demographic variables (age, gender, and level of education) and a possible learning effect which was operated by test presentation. All models were put through using the forward/backward best model selection. Each final model is presented with *R*^2^ and adjusted *R*^2^ for the model and β and sp^2^ values for each significant predictor. Where *R*^2^ shows how well the outcome value is explained by the model, β shows the change in the output variable associated with the predictor variable and sp^2^ shows the change in the output variable explained by the predictor variable. A *p*-value of <0.05 (2-tailed) was deemed significant.

Missing data for RAVLT short-term recall (paper-based version) were estimated using the Fully Conditional Specification (FSC) from the Mice library (Van Buuren and Groothuis-Oudshoorn, 2011). This estimate executed five imputations of the missing values and averaged them to return the user to the entire imputed matrix. The total sum of RAVLT trials and the long-term recall of RAVLT were used for such estimation. It was not possible to estimate missing data for the variable PASAT (digitized version) since reference variables to perform the estimation were not available. In addition, for the correlation and equivalence analysis variable transformation was performed when needed (i.e., reverse square root for long-term recall of RAVLT measures; reverse log10 for PASAT; and log10 transformation for TMT A and TMT B). Furthermore, for the regression analysis extreme values were removed using regression plots for the Cook distance (zResidual vs. Leverage). Only data without missing values was entered into the regression analysis.

## Results

The main demographic characteristics of the study sample (*N* = 81) are presented in Table 2. All participants presented a MMSE score over or equal to 26 (M = 28.98, SD = 1.05). Test presentation [the sequence of presenting first the traditional paper-based and second digitized tests (A) or vice versa (B)] is not completely equal due to participant withdrawal after study initiation.

The relationships between the traditional paper-based and digitized version of all studied test measures were analyzed using the non-parametric Spearman correlation test for the RAVLT Recognition, Corsi, Stroop Test and BNT-15 test measures and the parametric Pearson correlation test for all others (Table 3). The effect size for the correlation between the paper-based and digitized version of RAVLT learning and long-term recall, Corsi (both test measures), TMT (both test measures), Stroop Test Word-Color and BNT-15 test measures was large (effect size >0.50). Concerning the correlations between the paper-based and digitized versions of the RAVLT short-term recall and recognition, PASAT and Stroop Test Word and Interference test measures, the effect size was moderate (>0.30). The median correlation coefficient for all test measures had a large effect size with *r* = 0.53.

**TABLE 3:** Statistical comparison of paper-based and digitized versions of the test measures.

**Test**	**Measure**	**N**	***r***	**Mean (SD) paper-based**	**Mean (SD) digitized**	***t*-value difference**	***t*-value equivalence**
RAVLT	Learning	69	0.53^∗∗∗^	52.1 (10.6)	49.8 (11.2)		4.46^∗∗∗^
	STR	81	0.42^∗∗^	10.7 (2.6)	10.6 (3.4)		5.32^∗∗∗^
	LTR	77	0.59^∗∗∗^	10.7 (3.1)	10.6 (3.2)		5.96^∗∗∗^
	Recognition^†^	80	0.34^∗∗^	28.1 (2.1)	27.2 (3.2)	2.14^∗^	2.76^∗∗^
Corsi	Fwd. span^†^	77	0.59^∗∗∗^	5.9 (1.2)	5.7 (1.6)		4.46^∗∗∗^
	Bkwd. span^†^	74	0.53^∗∗∗^	5.7 (1.1)	5.3 (1.1)	2.28^∗^	3.76^∗∗∗^
PASAT	Correct no.	44	0.43^∗∗^	53.4 (6.0)	50.6 (9.2)		3.19^∗∗∗^
TMT	TMT A	78	0.62^∗∗∗^	28.9 (12.7)	30.3 (11.1)		5.46^∗∗∗^
	TMT B	78	0.53^∗∗∗^	68.2 (38.4)	71.2 (36.1)		5.71^∗∗∗^
Stroop Test	Word ^†^	78	0.45^∗∗∗^	19.0 (4.3)	28.6 (14.3)	5.72^∗∗∗^	
	Word-Colour ^†^	70	0.66^∗∗∗^	26.4 (7.57)	36.6 (13.1)	5.62^∗∗∗^	
	Interference ^†^	69	0.34^∗∗^	8.2 (5.9)	8.6 (10.8)		3.71^∗∗∗^
BNT-15	Correct no.^†^	79	0.67^∗∗∗^	13.1 (2.3)	11.0 (3.2)	4.64^∗∗∗^	

*^†^*r*_*s*_ (Spearman correlation coefficient), otherwise *r* (Pearson correlation coefficient). ^∗∗∗^*p* < 0.001; ^∗∗^*p* < 0.01; ^∗^*p* < 0.05. Not significant *r* and *t*-values are not displayed. STR, short-term recall; LTR, long-term recall; Fwd., forward; Bkwd., backward; no., number; TMT A, Trail Making Test part A; TMT B, Trail Making Test part B.*

Test scores were directly compared employing a *t*-test and an equivalence test (Table 3). For 8 out of 13 tests measures, the mean test scores were not statistically different from zero and were statistically equivalent to zero. This included test measures from RAVLT, Corsi, PASAT, TMT, and Stroop Test. Two test measures (RAVLT recognition and Corsi backward span) were statistically different from zero but were also statistically equivalent to zero, showing that while this difference was of statistical significance it was not of any practical significance, since it lay within the 1 SD equivalence borders. Three test measures (Stroop Word and Word-Color and BNT-15) were statistically different from zero and not statistically equivalent to zero.

Further, the effects of well-known variables affecting cognitive performance in the different tests measures were investigated. Linear regression models were carried out for each test measure and version (paper-based and digitized) including age, gender, level of education and test presentation as predictors. A best model forward/backward approach was employed, thus selecting the best combination of predictors (Table 4).

**TABLE 4:** Linear regression models of paper-based and digitized versions of the test measures.

**Test**	**Measure**	***R*^2^ adj. pp**	**Selected predictors pp**	***R*^2^ adj. app**	**Selected predictors app**
RAVLT	Learning	0.476	**a,t**	0.434	**a,t**
	STR	0.431	**a**,g,**t**	0.375	**a,t**
	LTR	0.341	**a,g**,t	0.401	**a,g**
	Recognition	0.345	**a,t**	0.308	**a**,**t**
Corsi	Fwd. span	0.473	**a**,g	0.472	**a**
	Bkwd. span	0.324	**a**,e	0.378	**a**,g
PASAT	Correct no.	0.249	**a**,t	0.429	**a**
TMT	TMT A	0.350	**a**	0.444	**a**,t
	TMT B	0.435	**a**,g	0.449	**a**,e,t
Stroop Test	Word	0.258	**a**	0.425	**a**
	Word-Color	0.354	**a**	0.540	**a**
	Interference	0.101	e	n.s.	
BNT-15	Correct no.	n.s.		n.s.	

**R*^2^ adj, explained adjusted variance; pp, paper-based test version; app, digitized test version; a, age; g, gender; e, level of education; t, test presentation; STR, short-term recall; LTR, long-term recall; Fwd., forward; Bkwd., backward; no., number; TMT A, Trail Making Test, part A; TMT B, Trail Making Test, part B; n.s., not significant. Bold case indicates overlapping predictors in the test versions.*

All but three regression models predicting the different test measures were significant (*p* < 0.05), both for paper-based and digitized versions (Table 4). The three exceptions were for the BNT-15, where neither the model for traditional paper-based nor the model for the digitized version were significant, and Stroop Test Interference, where only the model for traditional paper-based but not the model for the digitized version reached significance. All significant models are available in Supplementary Appendix B.

Concerning the individual effects of the demographic variables, age showed the strongest impact (0.44 ≤ | β| ≤ 0.74; 0.19 ≤ sp^2^ ≤ 0.55). In each test measure, except Stroop Interference, age was the strongest or only significant predictor in both test versions. A higher age consistently predicted a worse test result. Gender was the second demographic variable with the greatest influence on predicting cognitive test measures (0.20 ≤ | β| ≤ 0.35; 0.04 ≤ sp^2^ ≤ 0.12). Women outperformed men in the RAVLT test measures short-term recall in the paper-based test version (β = 0.24; sp^2^ = 0.06) and long-term recall for both test versions (β ≥ 0.22; sp^2^ ≥ 0.05). On the contrary, men outperformed women in Corsi forward span only in the paper-based version (β = −0.35; sp^2^ = 0.12), and Corsi backwards span only in the digitized version (β = −0.22; sp^2^ = 0.04) as well as in TMT B, but only in the traditional paper-based version (β = 0.20; sp^2^ = 0.04). A learning effect, operated by test presentation, was observed in several of the regression models (0.21 ≤ | β| ≤ 0.39; 0.04 ≤ sp^2^ ≤ 0.15). The effect of a second presentation of the test consistently predicted a better outcome and was a significant predictor in 7 out of 8 RAVLT test measures (0.21 ≤ | β| ≤ 0.39; 0.04 ≤ sp^2^ ≤ 0.15), paper-based version of PASAT (β = 0.35; sp^2^ = 0.12), and the digitized version of both TMT test measures (parts A and B) (β ≥ 0.21; sp^2^ ≥ 0.04). An education effect (0.20 ≤ | β| ≤ 0.34; 0.04 ≤ sp^2^ ≤ 0.11) was only found to be a significant predictor in Corsi backward span in the paper-based version (β = 0.23; sp^2^ = 0.05), TMT B in the digitized version (β = −0.20; sp^2^ = 0.04) and Stroop Test Interference measure in the paper-based version (β = −0.34; Δ*R*^2^ = 0.11), with a higher education consistently predicting a better test outcome. A learning effect (0.21 ≤ |β| ≤ 0.39; 0.04 ≤ sp^2^ ≤ 0.15) was observed for RAVLT, PASAT and TMT, with a second presentation of these tests in the different version resulting in a better test outcome. For detailed information of each and every model, please see Supplementary Appendix B.

## Discussion

The main focus of the study was to compare a new digitized cognitive screening, Minnemera, with its corresponding traditional paper-based cognitive tests. Construction of the self-administrative digitized tests closely resembled the construction of the traditional paper-based tests. Every test measure, one to four per test, was separately analyzed. The degree of compliance between test versions was assessed by calculating the correlation coefficients as well as the statistical difference and equivalence of the test scores between test versions. Results showed that every test in the paper-based cognitive screening was, at least moderately, correlated to its digitized version and that test scores, for those tests with small modifications, were statistically equivalent between the test versions. The impact of age, gender, level of education and learning effect was assessed with a multiple linear regression analysis. Age was the strongest predictor in all but one of the models, with an increase in age consistently predicting a decrease in test outcome. Some test measures were also affected by gender and/or level of education. A learning effect was observed for the RAVLT and to a lesser extent for PASAT and TMT (parts A and B). This study has been the first promising step in validating the digitized self-administrative cognitive screening battery Minnemera.

### Similarities and Differences Between Traditional Cognitive Tests and Their Corresponding Adaptation to Digitized Version

A correlation, corresponding to a moderate (>0.30) to large (>0.50) effect size (Cohen, 1988; Bosco et al., 2015), was found between the traditional and digitized versions in all test measures. Scores for all test measures, except for Stroop Test Word and Word-Color and BNT-15, were statistically equivalent (with a threshold of ±1 SD).

All four test measures of the digitized RAVLT were deemed comparable to the traditional paper-based version of the test. In constructing the digitized test, nearly no modifications were necessary. The effect sizes for the correlations ranged from moderate to large and every test measure showed to be statistically equivalent. A learning effect was observed for most of the RAVLT measures. This learning effect may have implied an amelioration of the results, thus a stronger correlation could have been expected when controlling for learning.

Similarly, the Corsi Block-tapping task, nearly an exact copy of the traditional paper-based version, is deemed comparable between test versions. Results on both test measures of the Corsi Block-tapping task demonstrated a correlation between the test versions with a large effect size, and overall statistical equivalence between test scores. These results are consistent with the previous study of eCorsi by Brunetti et al. (2014). Somewhat contrary to our findings, Claessen et al. (2015) showed only a small to moderate effect size, with higher accuracy for the forward traditional test than the forward digitized test, and comparable performance on the backward reproduction. These authors suggested that versions’ divergence could be explained as a result of motor priming and interference effects and, therefore, the underlying theoretical concept of the task needed further reconsideration in the digitized version.

Regarding the PASAT test, a moderate effect was shown when both versions were compared. The rate of non-response in this test was high, especially among the older participants with test presentation B (digitized test prior to traditional version). This indicates that the instructions and included practice for the digital test were possibly not sufficiently detailed for some participants to grasp what was expected of them during this test. This could further be explained by the comparatively large modifications necessary when adapting the original verbal PASAT test to the digitized non-verbal version (see section “Material”). This “non-verbal adaptation” could have included an additional element to the cognitive process of the participant’s final answer, extending the response time more than usual and thus affecting performance during the test. In any case, comparisons were carried out with participants who accomplished both test versions, obtaining a correlation with moderate effect size and statistical equivalence between the traditional and digitized test versions. To date, no other study has previously investigated the equivalence between a traditional and digitized version of the PASAT.

Our findings on TMT measures (TMT A and TMT B) showed a large effect size for the correlation between test versions. The test scores also showed statistical equivalence. These equivalent results were, to some extent, surprising considering that the traditional version required the use of a pencil and the digitized version the use of the participant’s index finger. This finding, however, is in line with previous comparable paper-based and computerized TMT studies (Fellows et al., 2017; Karimpoor et al., 2017). These results suggest that the digitized TMT can be considered comparable to the traditional paper-based version.

Furthermore, the effect size for the correlation between the traditional Stroop Test and its modified digitized version were moderate to large, which is in line with previous studies (Hepp et al., 1996; DiBonaventura et al., 2010). The digitized Stroop Test underwent minor modifications which mainly affected the scoring of this test. It is therefore not surprising that the results of the Word and Word-Colour tasks were found to be statistically different. The calculated Interference score, which was less dependent on the modifications, was found to be statistically equivalent between test versions.

Finally, the 15 items of the traditional BNT did not undergo any substantial modification in digitizing. The effect size for the correlation between the two test versions was large. However, the scores were statistically different with a lower score in the digitized version of the test. At present, no other study has investigated paper-based versus digitized visual confrontation naming tests.

Altogether, the findings addressed above are in line with other studies for Corsi (Brunetti et al., 2014), RAVLT (Morrison et al., 2018); Stroop Test (Hepp et al., 1996; DiBonaventura et al., 2010; and TMT (Fellows et al., 2017). Studies from the 1980s and 1990s showed greater differences between the different testing versions (Dillon, 1992), compared to later studies from the 2000s and onward (Noyes and Garland, 2008). These more recent studies claimed that differences between the traditional and the digitized test version had been reduced thanks to technological advances, and as the result of changing computer habits in everyday life. In addition, this gap reduction between traditional and digitized test methods could also be related to a change of focus in the studies. Earlier studies (from the 1980s and 1990s) mainly examined reading speed and reading comprehension, while recent studies investigate other aspects, such as the total outcome of a test, for example, or the total number of words recalled in a memory test (Noyes and Garland, 2008), in line with the focus of the present study.

### Influence of Age, Gender, Education and Learning Effect in the Different Versions and Test Measures

It is well-known that cognitive test results are affected by demographic variables, such as age, gender and level of education, as well as by learning, i.e., the effect of a second presentation of the same test (Leibovici et al., 1996; Salthouse, 1996; Jorm et al., 2004; Mungas et al., 2009; Scharfen et al., 2018). By means of a multiple linear regression analysis these effects were also studied in our sample. Age was the strongest predictor of all the studied test measures, with an increase in age associated with worse performance. Gender was the next strongest predictor, where a trend was observed of women outperforming men in language-related tests (RAVLT) and men outperforming women in tests dependent on spatial orientation (Corsi and TMT B). A weak positive tendency between level of education and mental flexibility was observed (TMT B and Stroop Test Interference). Lastly, a learning effect was observed in RAVLT, PASAT, and TMT.

The results of the RAVLT were influenced by age and gender, where an increase in age had a negative effect on test performance and where women performed somewhat better than men. These patterns are consistent with previous findings (Query and Megran, 1983; Geffen et al., 1990; Schmidt, 1996; Van der Elst et al., 2005; Strauss et al., 2006). Contrarily, educational level was not found to have an impact on the results of the RAVLT in the current study. Other studies have reported inconsistent results on the influence of education level on the results of the RAVLT (Mitrushina et al., 2005). The results of the Corsi Block-tapping task, both paper-based and digitized versions, were also adversely affected by an increase in age and a gender effect was observed where men outperformed women. These results are in line with other studies (Kessels et al., 2000). Increasing age also had a clear negative effect on the performance of PASAT, consistent with other research (Gronwall, 1977; Mitrushina et al., 2005; Ozakbas et al., 2016), but performance was not affected by either gender or level of education, which is further in line with what is reported in the literature (Deary and Der, 2005). The results of the TMT, both part A and part B, were also negatively affected by age which is in accordance with previous findings (Spreen and Strauss, 1998; Tombaugh, 2004; Mitrushina et al., 2005; Salthouse, 2011). Both Word and Word-color test measures of both the traditional paper-based Stroop Test and Minnemera’s digitized adaptation showed worse performance with increasing age, only the interference measure in the traditional paper-based version of the test was affected by education and none of the measures was affected by gender, which is all in line with previous research (Van der Elst et al., 2006; Hankee et al., 2016). As for the results on the BNT-15, no clear effect of age, gender or education was observed. In contrast to our findings, previous research has shown differences in performance on BNT depending on age, gender and level of education (Van Gorp et al., 1986; Neils et al., 1995). Van Gorp et al. (1986) found that there was a slight adverse effect on test performance with increasing age, but that it could be accounted for by the larger variances within the older age groups compared to the younger groups. Further, the conflicting results of the influence of educational level could be explained by the differences in the samples between the current study and Neils et al. (1995), in which subjects with low education (6–9 years) were included, and as a group obtained the poorest result on the Boston Naming Test. The differences between subjects with 10–12 years of education and > 12, were not as prominent compared to the group with low education.

Some limitations should be considered. The sample size was relatively small for stratification according to level of education, so the results concerning the effects of education should be considered as exploratory. Learning effect (operated by test presentations A and B) became somewhat distorted due to late dropouts, which potentially affected some test results. For example, results showed that at least PASAT was easier to implement for the group with condition A, who had been instructed by a test leader at the first test occasion. To overcome these limitations, further research should be carried out with larger sample sizes (Bates et al., 1996). Moreover, interpersonal reliability has not been considered in the current study. The influence of different test leaders could have had an effect on the test result. It is also one of the disadvantages of traditional testing (Noyes and Garland, 2008), that the risk of bias is greater, as the test situation and, which cannot be ignored, the assessment can also be affected by the test leader when administering the paper-based versions of the test. The clinical utility of a neuropsychological test is often determined by its sensitivity and specificity. This kind of analysis has not been possible in the current study, since such an analysis also requires a clinical sample. However, the sensitivity and specificity of the paper-version of all the included tests have been ascertained in numerous, previous studies (see section “Material”), which means that, given statistical comparability between the paper-based and digitized versions, satisfying sensitivity and specificity on good grounds can be assumed to apply to the digitized version as well.

While results of this study are in favor of comparability between the traditional paper-based tests and the new digitized tests, to some extent even an equivalence in test scores, it is impossible to construct a completely equivalent test in another medium. According to Dillon (1992) and Noyes and Garland (2008), it is not possible to achieve equivalent test versions since a paper-based and digitized test will partly involve different cognitive processes. However, the similarities and differences regarding underlying cognitive processes involved in testing with the different test versions were not examined in the present work and would be interesting to investigate in future studies. Moreover, digitized testing should never aim to replace a traditional cognitive assessment; rather, it should aim to make one part of the cognitive assessment, namely the cognitive screening, more reliable and easier to administer, and therefore available to more patients, which in turn would free up more time for clinicians to observe and interact with their patients.

## Conclusion

The results of this study support the fact that the digitized test battery Minnemera, to a relatively large extent, captures the same information about an individual’s cognitive state as the traditional tests upon which Minnemera is based. The tests were digitized, closely copying traditional paper-based tests and introducing the least amount of modifications, which was especially successful for RAVLT, Corsi, TMT, and BNT. This is reflected in the correlations with a moderate to large effect size observed for each and every test measure. Comparing scores between test versions, RAVLT, Corsi, PASAT, TMT and one Stroop Test measure showed statistically equivalent results. Together, these findings suggest that the digitized versions of RAVLT, Corsi, and TMT are acceptably comparable to the traditional paper-based test versions. For PASAT, Stroop and BNT-15, more research is needed. Previously well-documented demographic differences - implying a negative impact in cognitive performance due to older age, lower educational level, and in certain test measures, gender differences - were also found in the present study and followed similar patterns in both the traditional and the digitized test versions. This study has been the first step in validating the digital self-administered cognitive screening battery Minnemera. In order to further validate Minnemera the following studies are recommended: test-retest reliability and validation in a clinical population to investigate the battery’s sensitivity and specificity concerning detection of cognitive impairment.

## Data Availability Statement

The datasets generated for this study are available on request to the corresponding author.

## Ethics Statement

The study was reviewed and approved by the Regional Ethics Review Board in Stockholm (issue number 2017/2530-31). The participants provided their written informed consent to participate in this study.

## Author Contributions

AM and MB conceived the study conception and design. SB and WH performed the data collection supervised by AM. MB was responsible for analysis and interpretation of the data. SB, WH, and AM drafted the manuscript, with critical revision of ML.

## Conflict of Interest

AM and WH are staff of Mindmore AB, the company which has developed the Minnemera cognitive screening application. The remaining authors declare that the research was conducted in the absence of any commercial or financial relationships that could be construed as a potential conflict of interest.
