# Supplementary material for: Comparing Traditional and Digitized Cognitive Tests Used in Standard Clinical Evaluation – A Study of the Digital Application Minnemera
Source: Front Psychol. 2019 Oct 18;10:2327. doi: 10.3389/fpsyg.2019.02327 (PMC6813236; doi:10.3389/fpsyg.2019.02327)
Supplement: Supplementary file 1 [file Table_1.pdf]

Appendix A – Supplementary Methods

Adapted and modified version of traditional paper-based tests to digitized version

Rey Auditory Verbal Learning Test (RAVLT)

The RAVLT examines verbal learning and verbal memory (Rey, 1964; Schmidt, 1996). RAVLT has been widely disseminated and is available in several versions. The standard version is a list of 15 words (list A) which the test leader reads to the test person at a rate of one word per second. The test person then repeats as many words as she or he remembers. The procedure is repeated five times (trials 1 to 5), after which a new list of 15 new words (list B), so-called distractors, are presented with only one attempt to repeat them is given (trial 6). Immediately afterwards, the test person may repeat as many words as possible from the first list (without getting them read again, trial 7). After 20-30 minutes, the procedure is repeated, and the test person is asked to repeat as many words as possible from the first list (trial 8). Subsequently, the test person will hear a list of the words from both list A and list B and the task is to answer which of the words were included on the first list (recognition trial). The digitized test is designed in the same way as above. Both versions of the RAVLT have four separate outcome measures. Learning curve is scored as the total of correctly recalled words trials 1 to 5. Short term memory is scored as the total of correctly recalled words in trial 7. Long term memory is scored as the total of correctly recalled words in trial 8. Recognition is scored as the total of correctly recognised words (true positives and true negatives) in the recognition trial. Data in the digitized version is collected through speech recognition and underwent both an automatic scoring step and a manual correction of the transcripts (e.g. recognising “färghus” (= colourhouse) as the two separate words “färg” and “hus” (= colour and house)). This manual correction has been used to improve the automatic scoring algorithm. Otherwise, scoring is equal for both test versions.

Corsi block-tapping test (Corsi)

This test was used to assess visuospatial memory (Corsi, 1972). The test consists of nine blocks and the test leader demonstrates a sequence of different numbers of blocks. The sequence begins with two blocks in a sequence that the test person is asked to repeat. The difficulty level rises to a maximum of nine blocks until the test person reproduces the wrong sequence twice in a row. The second part of the test is to repeat the same series of sequences but backwards. There are different versions of the Corsi, but in the traditional test that was used in the current study, the test leader showed a sequence with a finger on a white paper with black cubes. In the digitized version, the

background is black and the boxes are white, and the boxes in the sequence blink, one at a time. The maximum sequence correctly responded to by the participant in either part (forward and backward) were scored as the outcome measures in both test versions.

Paced Auditory Serial Addition Test (PASAT)

PASAT was used to assess information processing speed and attention. The test person hears a number every three seconds and is asked to add the number they have just heard with the previous digit. The test involves working memory, concentration, attention and arithmetic skill. In the original study (Gronwall, 1977) a new digit was presented at 2.4 second intervals and there are also studies with 2 seconds between the figures, but in the current study, the figures were presented at 3 second intervals (Rudick, Antel, Confavreux, Cutter, Ellison, Fischer, et al., 1997). In contrast to the paper-based test, where the participant responds verbally to the sum of two consecutive numbers they hear, the digitized version in the Minnemera application was designed to respond non-verbally to the representation of that calculation by means of two button options “odd” or “even”. The amount correct responses was scored as the outcome measure for both test versions. The current study used norms in accordance with Spreen and Strauss (1998).

Trail Making Test (TMT)

The original version of the TMT was developed by the United States Army (Army Individual Test battery, 1944). The test has two parts: TMT A, examines visual scanning capability and cognitive process speed, while TMT B measures mental flexibility (Tombaugh, 2004). Each part consists of 25 circles on a paper. In part A, the circles are numbered from 1 to 25. The test person is instructed to draw a line between them in ascending order of numbers, from 1 to 25, as rapidly as possible. Part B consists of 13 digits and 12 letters to be paired and the test person is instructed to draw a dash between them in ascending number and letter order (1-A, 2-B, 3-C and so on). In the traditional version, originally developed by the US Army, paper and pencil were used. The digital adaptation required to turn the original A4 sheet 90 degrees left to fit into the horizontal 10.1” screen of the tablet in a similar way. Thus, even though number position were not modified, their location in the screen’s canvas was shifted compared to the original paper presentation. Moreover, the digitized version was performed with their index finger instead of a pen. Total time for completion was scored as the outcome measure in both test versions. The norms used in the current study were based on Tombaugh (2004).

Stroop Test

The Stroop test (Stroop, 1935) measures executive function and, more specifically, the ability to inhibit a learned response for the benefit of another (Spreen and Strauss, 1998). In the Victoria

Stroop test (Regard, 1991), used in the current study, three different tasks are performed, each task with 24 dots, 24 neutral words or 24 words that indicate a colour which can be printed in another colour, that is, in this way incongruent. The test person should as rapidly as possible say the colour with which the word is written, not the colour that the colour name says. Here the total time for completion is scored as the outcome measure per task (dot, word and word-colour). The interference outcome measure was calculated by subtracting the word outcome measure from the word-colour outcome measure. In the digitized test, participants underwent two tasks: reading of neutral (congruent) words and incongruent words. However, the participants could see only one word at a time, unlike in the traditional test where all 24 words were introduced on the same paper. The participants saw a word in the digitized version and had to press one of four boxes (green, yellow, blue or red), to indicate which of the colours matched the given word. In this version the average response time for correct responses was calculated as the outcome measure per task (word and word-colour). As in the traditional paper-based version, the interference outcome measure was calculated by subtracting the word outcome measure from the word-colour outcome measure. Thus, in both test versions, the ability to inhibit a learned response was tested. The standards for the Stroop test were used according to Regard (1991).

Boston Naming Test (BNT)

BNT is a naming test and aims to examine semantic memory (Jorgensen, Johannesen & Vogel, 2017; Kaplan, Goodglass & Weintraub, 1983). The complete test consists of 60 images of different objects, while the short form used in the current study consists of 15 images (BNT-15), which is a modified variant of Jorgensen et al. (2017). The task is to name the objects in the pictures. Administration time is a maximum of 20 seconds per image. Points are only given if the test person indicates the correct answer within 20 seconds. The digitized test in the Minnemera application was designed in the same way as above. The amount correct responses is scored as the outcome measure in both test versions. Only in the digitized version where data is collected through speech recognition, scoring first proceeded through an automatic scoring step and subsequently a manual correction of the transcripts. This manual correction has been used to improve the automatic scoring algorithm. Norms for a Danish population were used in the current study (Jorgensen et al., 2017).

Appendix B – Supplementary Tables

Table 1a. Multiple regression analysis for RAVLT Learning traditional paper-based version

	R^2	R^2 adj	B (SE)	β	*p* (β)	sp^2	*p* (sp^2)
	0.490	0.476					
Constant			56.93 (3.28)		<0.001		
Age			-0.33 (0.04)	-0.64	<0.001	0.40	<0.001
Test presentation			8.02 (1.72)	0.39	<0.001	0.15	0.001

Table 1b. Multiple regression analysis for RAVLT Learning digitized version

	R^2	R^2 adj	B (SE)	β	*p* (β)	sp^2	*p* (sp^2)
	0.450	0.434					
Constant			70.31 (2.98)		<0.001		
Age			-0.34 (2.04)	-0.60	<0.001	0.36	<0.001
Test presentation			-5.41 (2.04)	-0.24	0.010	0.06	0.047

Table 1c. Multiple regression analysis for RAVLT STR traditional paper-based version

	R^2	R^2 adj	B (SE)	β	*p* (β)	sp^2	*p* (sp^2)
	0.453	0.431					
Constant			10.9 (1.03)		<0.001		
Age			-0.08 (0.01)	-0.64	<0.001	0.39	<0.001
Gender			1.25 (0.04)	0.24	0.006	0.06	0.036
Test presentation			1.46 (0.04)	0.29	0.001	0.08	0.012

Table 1d. Multiple regression analysis for RAVLT STR digitized version

	R^2	R^2 adj	B (SE)	β	*p* (β)	sp^2	*p* (sp^2)
	0.392	0.375					
Constant			15.95 (0.80)		<0.001		
Age			-0.08 (0.01)	-0.52	<0.001	0.26	<0.001
Test presentation			-1.47 (0.55)	-0.25	0.009	0.06	0.034

Table 1e. Multiple regression analysis for RAVLT LTR traditional paper-based version

	R^2	R^2 adj	B (SE)	β	*p* (β)	sp^2	*p* (sp^2)
	0.367	0.341					
Constant			11.04 (1.19)		<0.001		
Age			-0.08 (0.01)	-0.59	<0.001	0.32	<0.001
Gender			1.48 (0.53)	0.27	0.007	0.07	0.024
Test presentation			1.31 (0.52)	0.24	0.014	0.05	0.042

Table 1f. Multiple regression analysis for RAVLT LTR digitized version

	R^2	R^2 adj	B (SE)	β	*p* (β)	sp^2	*p* (sp^2)
	0.417	0.401					
Constant			13.88 (1.03)		<0.001		
Age			-0.09 (0.01)	-0.64	<0.001	0.40	<0.001
Gender			1.21 (0.51)	0.22	0.021	0.05	0.069

Table 1g. Multiple regression analysis for RAVLT Recognition traditional paper-based version

	R^2	R^2 adj	B (SE)	β	*p* (β)	sp^2	*p* (sp^2)
	0.362	0.345					
Constant			29.54 (0.66)		<0.001		
Age			-0.05 (0.01)	-0.57	<0.001	0.32	<0.001
Test presentation			1.05 (0.35)	0.28	0.003	0.08	0.014

Table 1h. Multiple regression analysis for RAVLT Recognition digitized version

	R^2	R^2 adj	B (SE)	β	*p* (β)	sp^2	*p* (sp^2)
	0.327	0.308					
Constant			32.70 (0.95)		<0.001		
Age			-0.06 (0.01)	-0.50	<0.001	0.25	<0.001
Test presentation			-1.06 (0.49)	-0.21	0.034	0.04	0.074

Table 2a. Multiple regression analysis for Corsi Forward Span traditional paper-based version

	R^2	R^2 adj	B (SE)	β	*p* (β)	sp^2	*p* (sp^2)
	0.487	0.473					
Constant			8.39 (0.34)		<0.001		
Age			-0.03 (<0.01)	-0.55	<0.001	0.29	<0.001
Gender			-0.70 (0.17)	-0.35	<0.001	0.12	0.003

Table 2b. Multiple regression analysis for Corsi Forward Span digitized version

	R^2	R^2 adj	B (SE)	β	*p* (β)
	0.479	0.472			
Constant			8.33 (0.31)		<0.001
Age			-0.4 (0.01)	-0.69	<0.001

Table 2c. Multiple regression analysis for Corsi Backward Span traditional paper-based version

	R^2	R^2 adj	B (SE)	β	*p* (β)	sp^2	*p* (sp^2)
	0.342	0.324					
Constant			5.83 (0.65)		<0.001		
Age			-0.03 (0.01)	-0.49	<0.001	0.22	<0.001
Education			0.64 (0.28)	0.23	0.025	0.05	0.061

Table 2d. Multiple regression analysis for Corsi Backward Span digitized version

	R^2	R^2 adj	B (SE)	β	*p* (β)	sp^2	*p* (sp^2)
	0.396	0.378					
Constant			7.44 (0.36)		<0.001		
Age			-0.03 (0.01)	-0.53	<0.001	0.26	<0.001
Gender			-0.44 (0.20)	-0.22	0.033	0.04	0.088

Table 3a. Multiple regression analysis for PASAT traditional paper-based version

	R^2	R^2 adj	B (SE)	β	*p* (β)	sp^2	*p* (sp^2)
	0.274	0.249					
Constant			53.32 (2.35)		<0.001		
Age			-0.12 (0.03)	-0.44	<0.001	0.19	0.001
Test presentation			3.91 (1.24)	0.35	0.003	0.12	0.006

Table 3b. Multiple regression analysis for PASAT digitized version

	R^2	R^2 adj	B (SE)	β	*p* (β)
	0.446	0.429			
Constant			61.67 (1.61)		<0.001
Age			-0.19 (0.04)	-0.67	<0.001

Table 4a. Multiple regression analysis for TMT A traditional paper-based version

	R^2	R^2 adj	B (SE)	β	*p* (β)
	0.358	0.350			
Constant			8.49 (3.2)		0.010
Age			0.37 (0.1)	0.60	<0.001

Table 4b. Multiple regression analysis for TMT A digitized version

	R^2	R^2 adj	B (SE)	β	*p* (β)	sp^2	*p* (sp^2)
	0.459	0.444					
Constant			6.99 (3.13)		0.029		
Age			0.28 (0.04)	0.59	<0.001	0.33	0.001
Test presentation			4.90 (1.68)	0.26	0.005	0.07	0.029

Table 4c. Multiple regression analysis for TMT B traditional paper-based version

	R^2	R^2 adj	B (SE)	β	*p* (β)	sp^2	*p* (sp^2)
	0.450	0.435					
Constant			1.04 (9.14)		0.910		
Age			0.80 (0.12)	0.60	<0.001	0.34	<0.001
Gender			11.02 (4.87)	0.20	0.027	0.04	0.091

Table 4d. Multiple regression analysis for TMT B digitized version

	R^2	R^2 adj	B (SE)	β	*p* (β)	sp^2	*p* (sp^2)
	0.473	0.449					
Constant			37.14 (13.62)		0.008		
Age			0.63 (0.10)	0.56	<0.001	0.30	<0.001
Test presentation			9.20 (4.03)	0.21	0.026	0.04	0.095
Education			-11.93 (5.49)	-0.20	0.033	0.04	0.113

Table 5a. Multiple regression analysis for Stroop Word traditional paper-based version

	R^2	R^2 adj	B (SE)	β	*p* (β)
	0.260	0.258			
Constant			13.49 (1.06)		<0.001
Age			0.10 (0.02)	0.52	<0.001

Table 5b. Multiple regression analysis for Stroop Word digitized version

	R^2	R^2 adj	B (SE)	β	*p* (β)
	0.434	0.425			
Constant			16.11 (1.22)		<0.001
Age			0.16 (0.02)	0.66	<0.001

Table 5c. Multiple regression analysis for Stroop Word-Colour traditional paper-based version

	R^2	R^2 adj	B (SE)	β	*p* (β)
	0.363	0.354			
Constant			16.46 (1.57)		<0.001
Age			0.18 (0.03)	0.60	<0.001

Table 5d. Multiple regression analysis for Stroop Word-Colour digitized version

	R^2	R^2 adj	B (SE)	β	*p* (β)
	0.548	0.540			
Constant			17.62 (1.81)		<0.001
Age			0.29 (0.04)	0.74	<0.001

Table 5e. Multiple regression analysis for Stroop Interference traditional paper-based version

	R^2	R^2 adj	B (SE)	β	*p* (β)
	0.113	0.101			
Constant			1.85 (0.15)		<0.001
Education			-0.24 (0.08)	-0.34	0.003
